# *Salmonella* Enteritidis Infections, United States, 1985–1999

**DOI:** 10.3201/eid1001.020572

**Published:** 2004-01

**Authors:** Mary E. Patrick, Penny M. Adcock, Thomas M. Gomez, Sean F. Altekruse, Ben H. Holland, Robert V. Tauxe, David L. Swerdlow

**Affiliations:** * Centers for Disease Control and Prevention, Atlanta, Georgia, USA; † U.S. Department of Agriculture, Atlanta, Georgia, USA; ‡ Food and Drug Administration, Rockville, Maryland, USA

**Keywords:** Salmonella Enteritidis, eggs, outbreaks

## Abstract

*Salmonella enterica* serotype Enteritidis emerged as an important illness during the 1980s. Investigations showed that consumption of undercooked eggs was the major risk factor for disease, and a variety of prevention and control efforts were initiated during the 1990s. We describe sporadic infections and outbreaks of *S.* Enteritidis in the United States from 1985 through 1999 and discuss prevention and control efforts. After reaching a high of 3.9 per 100,000 population in 1995, *S.* Enteritidis infections declined to 1.98 per 100,000 in 1999. While the total number of outbreaks decreased by half, those in the western states tripled. Outbreaks of *S.* Enteritidis phage type 4 infections accounted for 49% of outbreaks in 1999. Outbreak-associated deaths in health facilities decreased from 14 in 1987 to 0 in 1999. Overall, rates of sporadic *S.* Enteritidis infection, outbreaks, and deaths have declined dramatically. For further reductions, control measures should continue to be applied along the entire farm-to-table continuum.

*Salmonella* *enterica* serotype Enteritidis is one of the most common *Salmonella* serotypes worldwide, particularly in developed countries. During the 1980s, *S.* Enteritidis emerged as an important cause of human illness in the United States. In 1976, the incidence of *S.* Enteritidis was 0.55 per 100,000 population and represented only 5% of all *Salmonella* isolates. By 1985, this proportion reached 10%, and the rate increased to 2.4 per 100,000 population (*1*). During the same time, total *Salmonella* infection rates rose from 10.7 per 100,000 in 1976 to 24.3 in 1985. The highest rates of *S.* Enteritidis were seen in the Northeast, although rates in the western region also increased during this time.

 The number of outbreaks of *S.* Enteritidis infection also increased during the 1980s (*2*), particularly in the northeastern United States. Laboratory subtyping of *S.* Enteritidis isolates from outbreaks indicated that phage types (PT) 8 and 13a were the most common phage types in the United States (*3*). Although PT4 was common in Europe, where it coincided with a large increase in *S.* Enteritidis infections (*4*,*5*), it was seen in the United States only among persons with a history of foreign travel.

Case-control studies of sporadic *S.* Enteritidis infections and outbreaks demonstrated that shell eggs were the major risk factor for disease (*2*,*6*,*7*). After the implicated eggs were traced back to the farm of origin, microbiologic surveys showed *S.* Enteritidis of the same phage type that caused human cases to be present in the farm environment of egg-layer poultry flocks (*8*–*10*). Studies showed that the internal contents of eggs can be contaminated with *S.* Enteritidis (*11*,*12*), and this contamination has been identified as a major risk factor in the emergence of human illness. To reduce *S.* Enteritidis in eggs, on-farm prevention and control measures and quality assurance programs were initiated in the early 1990s. Education of consumers and food workers regarding the risks of consuming raw or undercooked eggs was also begun, with special emphasis on high-risk populations such as the elderly, children, pregnant women, and others with weakened immune systems. Restaurants and health institutions were encouraged to avoid pooling eggs, to use pasteurized egg product, and to avoid raw egg recipes. Requirements for refrigeration during distribution and storage were increased.

 We examined trends in *S.* Enteritidis infection in the United States from 1985 through 1999 based on surveillance data for sporadic infections and outbreaks reported to the Centers for Disease Control and Prevention (CDC). We describe prevention and control efforts and suggest a plan for further reduction of *S.* Enteritidis infections.

## Methods

 The CDC National Salmonella Surveillance System is a laboratory-based passive system that was developed in 1976. In 1994, most states began reporting cases electronically to this system through the Public Health Laboratory Information System (*13*). Annual *S.* Enteritidis isolation rates per 100,000 population were calculated for each state and region of the United States by using 1976–1999 Census data.

 Before 1985, reports of *S.* Enteritidis outbreaks were collected through the National Foodborne Disease Outbreak Surveillance System. In response to a growing number of outbreaks and the need for timely follow-up, CDC began the *S.* Enteritidis Outbreak Reporting System in 1985. This system encouraged officials from state and local health departments to report outbreaks as soon as they occurred. At the end of each year, epidemiologists from state health departments were asked to verify the information that CDC had received and to send written reports of additional outbreaks. An outbreak of *S.* Enteritidis infection was defined as >2 cases of laboratory-confirmed *S.* Enteritidis infection in persons who ingested a common food, or one culture-confirmed case with additional cases meeting a clinical definition of illness and *S.* Enteritidis isolated from a food specimen. Other information collected about each outbreak included the city, county, state, location where the food was prepared, and location where the food was consumed. The total number of outbreak-associated cases included all symptomatic persons with either culture-confirmed or epidemiologically linked infection.

 Outbreak-associated foods were considered to be confirmed vehicles of transmission if 1) they were statistically implicated in an epidemiologic study, 2) *S.* Enteritidis was isolated from leftover foods, or 3) if the food item was the only food consumed by all ill persons (this occurred in <10 outbreaks). If eggs were implicated or were a primary ingredient in the implicated food, the outbreak was classified as egg-associated.

 A subset of isolates from patients, food workers, implicated foods and farm specimens associated with outbreaks were phage typed at CDC and the U.S. Department of Agriculture (USDA) using a technique described by Ward et al. (*14*). Regions of the United States were defined as follows: Northeast—Connecticut, Maine, Massachusetts, New Hampshire, New Jersey, New York, Pennsylvania, Rhode Island, and Vermont; Midwest—Illinois, Indiana, Iowa, Kansas, Michigan, Minnesota, Missouri, Nebraska, North Dakota, Ohio, South Dakota, and Wisconsin; South—Alabama, Arkansas, Delaware, the District of Columbia, Florida, Georgia, Kentucky, Louisiana, Maryland, Mississippi, North Carolina, Oklahoma, South Carolina, Tennessee, Texas, Virginia, and West Virginia; and West—Alaska, Arizona, California, Colorado, Hawaii, Idaho, Montana, Nevada, New Mexico, Oregon, Utah, Washington, and Wyoming.

## Results

### National *Salmonella* Surveillance Data

 In the late 1980s and early 1990s, the proportion of *Salmonella* isolates that were *S.* Enteritidis continued to rise, and in 1994, it became the most common *Salmonella* serotype in the United States, representing 26% of all *Salmonella* isolates. Since then, however, the proportion has steadily decreased, reaching 16% in 1999.

**Figure 1:**
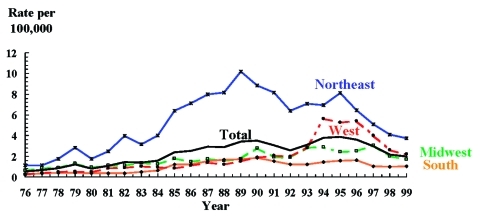
*Salmonella* Enteritidis isolation rates per 100,000 population, by selected regions,* United States, 1976–1999. *Northeast: Connecticut, Maine, Massachusetts, New Hampshire, New Jersey, New York, Pennsylvania, Rhode Island, Vermont; Midwest: Illinois, Indiana, Iowa, Kansas, Michigan, Minnesota, Missouri, Nebraska, North Dakota, Ohio, South Dakota, Wisconsin; South: Alabama, Arkansas, Delaware, District of Columbia, Florida, Georgia, Kentucky, Louisiana, Maryland, Mississippi, North Carolina, Oklahoma, South Carolina, Tennessee, Texas, Virginia, West Virginia; and West: Alaska, Arizona, California, Colorado, Hawaii, Idaho, Montana, Nevada, New Mexico, Oregon, Utah, Washington, Wyoming.

 The *S.* Enteritidis incidence rate increased from 2.38 per 100,000 population in 1985 to 3.9 per 100,000 in 1995. Since then, there has been a decline of 49%, to 1.98 per 100,000 in 1999 (Figure 1). This decline mirrors that of total *Salmonella* infections, which fell 51% to 12.0 per 100,000 in 1999. Rates of *S.* Enteritidis infection in the Northeast showed the greatest change, increasing from 6.39 per 100,000 in 1985 to 10.2 in 1989 and then dropping 63% to 3.8 per 100,000 in 1999 (Figure 1). Rates in the West rose from 0.87 per 100,000 in 1985 to 5.6 per 100,000 in 1994, and then fell to 2.2 per 100,000 in 1999, a decline of 61%. Rates in the Midwest rose from 1.81 per 100,000 in 1985 to 3.1 in 1997 and then decreased to 1.7 in 1999, while the rate in the South rose from 1.22 per 100,000 in 1985 to 1.85 in 1990, and then fell to 1.04 in 1999.

### Outbreak Surveillance

**Figure 2:**
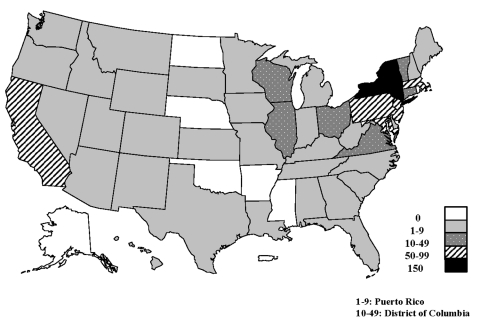
Reported outbreaks of *Salmonella* Enteritidis outbreaks, by state, 1985–1999 (N = 841). Includes two multistate outbreaks.

 From 1985 through 1999, a total of 841 outbreaks of *S.* Enteritidis infection were reported to CDC, affecting residents of 43 states, the District of Columbia, and Puerto Rico (Figure 2). The number of reported outbreaks increased from 26 in 1985 to 85 in 1990. Since 1990, outbreaks have declined 48%, to 44 in 1999. Reported outbreaks affected 29,762 persons; 2,904 (10%) were hospitalized (range 4%-21%), and 79 (0.3%) patients died (range 0%-0.9%) (Table 1). The median number of cases per outbreak decreased from 24 in 1985 to 10 in 1998.

**Table 1:** Foodborne outbreaks of *Salmonella* serotype Enteritidis infection, United States, 1985–1999

	All outbreaks (N = 841)						Outbreaks in health care facilities^a^ (N=89)			
Y	No. outbreaks	No. Ill	Median no. cases	No. hosp (%)	No. deaths (%)	No. outbreaks		No. Ill	No. (%) hospitals	No. deaths (%)	
1985	26	1159	24.0	144(12)	1 (.08)	3(12)		55	10(18)	1(2)	
1986	47	1444	12.0	107 (7)	6 (.4)	7(15)		105	10(10)	5(5)	
1987	58	2616	17.5	557(21)	15 (.6)	8(14)		489	391(80)	14(3)	
1988	48	1201	12.5	155(13)	11 (.9)	7(15)		131	2(2)	9(7)	
1989	81	2518	23.0	206 (8)	15 (.6)	19(23)		505	34(7)	13(3)	
1990	85	2656	18.0	318(12)	3 (.1)	12(14)		303	22(7)	3(1)	
1991	74	2461	15.0	200 (8)	5 (.2)	8(11)		118	6(5)	4(3)	
1992	63	2348	13.0	233(10)	4 (.2)	2 (3)		42	2(5)	2(5)	
1993	66	2215	16.5	219(10)	6 (.3)	5 (8)		56	4(7)	4(7)	
1994	51	5492	14.0	214 (4)	0	0		0	0	0	
1995	56	1312	12.0	113 (9)	8 (.6)	8(14)		156	21(13)	7(4)	
1996	47	1414	12.0	158(11)	2 (.1)	3 (6)		64	9(14)	0	
1997	46	1102	13.0	124(11)	0	1 (2)		13	1(8)	0	
1998	49	744	10.0	93(13)	3 (.4)	3 (6)		32	6(19)	3(9)	
1999	44	1080	15.0	63 (6)	0	3 (7)		12	5(42)	0	
Total	841	29,762	15.0	2,904 (10)	79 (.3)	89 (11)		2,081	523 (25)	65(3)	

^a^N=841. ^b^Includes hospitals (14), nursing/extended care homes (69), assisted/independent living facilities (2), and drug/alcohol rehabilitation facilities (4)

**Figure 3:**
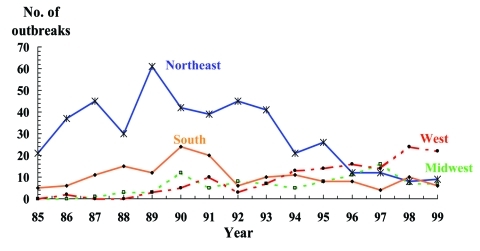
*Salmonella* Enteritidis outbreaks by region, United States, 1985–1999. Northeast: Connecticut, Maine, Massachusetts, New Hampshire, New Jersey, New York, Pennsylvania, Rhode Island, Vermont; Midwest: Illinois, Indiana, Iowa, Kansas, Michigan, Minnesota, Missouri, Nebraska, North Dakota, Ohio, South Dakota, Wisconsin; South: Alabama, Arkansas, Delaware, District of Columbia, Florida, Georgia, Kentucky, Louisiana, Maryland, Mississippi, North Carolina, Oklahoma, South Carolina, Tennessee, Texas, Virginia, West Virginia; and West: Alaska, Arizona, California, Colorado, Hawaii, Idaho, Montana, Nevada, New Mexico, Oregon, Utah, Washington, Wyoming.

 Although the total number of outbreaks of *S.* Enteritidis infection declined from 1985 to 1999, regional rates have shifted dramatically. In the Northeast region, outbreaks decreased from 21 (81%) of 26 outbreaks in the United States in 1985 to 9 (20%) of 44 outbreaks in 1999 (Figure 3). Conversely, outbreaks in the Western region increased from 0 in 1985 to 22 (50%) of 44 outbreaks in 1999. Most of these outbreaks occurred in California, where the percentage of outbreaks increased from 0 in 1986 to 16 (73%) of the 22 Western region outbreaks in 1999. The percentage of outbreaks in the Midwestern region averaged 9% (range 0%-35%), while the percentages of outbreaks in the South averaged 10% (range 9%-31%).

### Outbreak Settings

 Five hundred twenty-two (62%) outbreaks of *S.* Enteritidis infection were associated with food prepared at commercial food establishments (restaurants, caterers, delicatessens, bakeries, cafeteria, or market), 112 (13%) were associated with food prepared in a private home, 55 (7%) with food prepared at schools or churches, and 20 (2%) with food served in prisons. Forty-three (5%) outbreaks involved foods prepared at other locations, such as camps, cruise ships, workplace, shelter, festivals, or an unknown location. Eighty-nine (11%) outbreaks involved food served to residents of hospitals or nursing homes.

**Figure 4:**
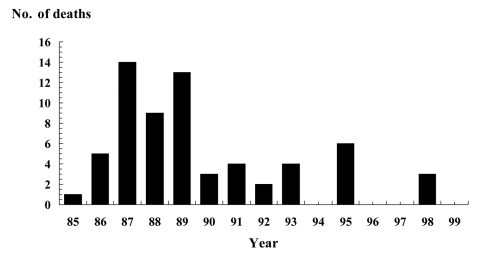
Deaths associated with outbreaks of *Salmonella* Enteritidis infections in healthcare facilities, 1985–1999 (N = 64).

 Of 79 outbreak-associated deaths, 65 (82%) were among persons in healthcare facilities (55 deaths in nursing or extended-care homes and 10 deaths in hospitals) (Table 1). Deaths in healthcare facilities decreased from 14 in 1987 to 0 in 1994, 1996, 1997, and 1999 (Figure 4). Overall, the outbreak-associated case-fatality rate in healthcare institutions was 3% (range 0%-9% per year), higher than the average case-fatality rate of 0.3% for all outbreaks. The proportion of outbreaks occurring at healthcare institutions between 1992 and 1999 ranged from 2% to 14%, noticeably lower than the 11%-23% of outbreaks occurring at these facilities from 1985 through 1991.

### Outbreak Vehicles

 A food item was implicated in 389 (46%) outbreaks of *S.* Enteritidis infection from 1985 through1999; in 86 (22%) of these, more than one food item was implicated. Of the 371 outbreaks for which information was available, 298 (80%) were egg associated. This proportion ranged from 10 (71%) of 14 in 1985 to 19 (95%) of 20 in 1997 (Table 2). Of outbreaks caused by a single vehicle for which information was known, 243 (83%) of 294 were egg-associated, as were 55 (71%) of 77 outbreaks in which more than one food item was implicated.

**Table 2:** Reported outbreaks of *Salmonella* Enteritidis infection with confirmed vehicle that contained eggs as a principal ingredient, by year

	No. of outbreaks	Outbreaks with confirmed vehicle No. (%)	Outbreaks with a confirmed vehicle that contained eggs^a^ No. (%)
1985	26	14(54)	10(71)
1986	47	22(47)	15/20(75)
1987	58	28(48)	21/24(88)
1988	48	25(52)	20/24(83)
1989	81	30(37)	19/28(68)
1990	85	30(35)	24/30(80)
1991	74	29(39)	20/26(77)
1992	63	35(56)	31/33(94)
1993	66	40(61)	31(78)
1994	51	29(57)	22(76)
1995	56	22(39)	15(68)
1996	47	26(55)	21/25(84)
1997	46	22(48)	19/20(95)
1998	49	18(37)	15/17(88)
1999	44	19(43)	15(79)
Total	841	389(46)	298/371(80)

 Among single foods implicated in egg-associated outbreaks, 67 (28%) of 243 were foods that contained raw eggs (e.g., homemade ice cream, Caesar salad dressing, tiramisu, egg nog). Sixty-five (27%) of the outbreaks implicated traditional egg dishes such as omelets, French toast, pancakes, and foods that use egg batter, such as crab cakes, chile rellenos, egg rolls, and Monte Cristo sandwiches. Sixty-three (26%) outbreaks implicated dishes known to contain eggs, such as lasagna, ziti, and stuffing, which would have been expected to have been fully cooked but probably did not reach temperatures sufficient to kill *S.* Enteritidis. Thirty-six (15%) outbreaks implicated egg dishes that were “lightly cooked” (e.g., hollandaise sauce, meringue, cream pies). The food vehicles in 12 (5%) outbreaks were reported to contain eggs but could not be classified because information on how the dishes were prepared was not provided.

 Seventy-three (20%) of the 371 confirmed outbreaks for which information was provided involved vehicles that did not contain eggs. Twenty (27%) of these outbreaks were associated with poultry (chicken or turkey), 8 (11%) with beef, and 6 (8%) with foods containing shrimp (3 outbreaks), bologna (1), pork (1), and pepper loaf (1). Other implicated foods included potatoes (3), beans (3), desserts (3), salad (3), macaroni and cheese (1), cheese sauce (1), goat cheese (1), chili (1), and a pureed diet (1). In 22 (30%) of the non–egg-associated outbreaks, more than one food was implicated. In four of these outbreaks, cross-contamination with raw eggs was suspected.

### Phage Types

 From 1988 through 1999, isolates from 455 outbreaks were submitted to CDC for phage typing. A single phage type was implicated in 436 (96%) of these outbreaks; 186 (43%) were caused by PT8, 96 (22%) by PT13a, and 64 (15%) by PT4. Other phage types included PT13 (20 outbreaks), PT34 (14 outbreaks), PT2 (13 outbreaks), and PT14b (9 outbreaks). Phage types differed by geographic region. In the Northeast and South, PT8 was the most common cause of *S.* Enteritidis outbreaks, followed closely by PT13a. Both PT8 and PT13a were common in the Midwest, while PT4 was predominant in the Western region.

**Figure 5:**
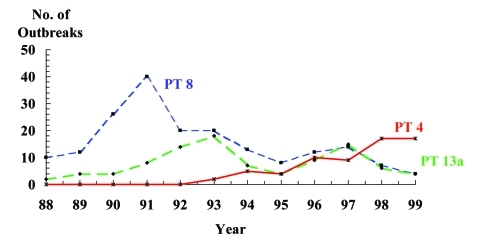
Frequency of outbreaks of *Salmonella* Enteritidis infection, by phage type, United States, 1988–1999 (N = 346). Phage types were not collected until 1988.

 The predominant phage types associated with *S.* Enteritidis outbreaks changed from 1988 through 1999. The proportion of outbreaks caused by PT8 and PT13a has decreased, while PT4 outbreaks have increased (Figure 5). In 1993, PT4 accounted for 2 (4%) of 47 *S.* Enteritidis outbreaks, while in 1999, PT4 represented 17 (49%) of 35 *S.* Enteritidis outbreaks and was the most common phage type. Most PT4 outbreaks were in the Western region; 52 (81%) of the 64 *S.* Enteritidis PT4 outbreaks reported from 1993 through 1999 occurred in California.

 Hospitalization and death rates did not differ by phage type. For all years combined, hospitalization rates were 180 (9%) per 1,899 cases in PT4 outbreaks, 694 (10%) per 6,910 cases in PT8 outbreaks, and 267 (12%) per 2,281 cases in PT3a outbreaks. Death rates were not statistically different among phage types: 0.16% in PT4 outbreaks, 0.38% in PT8 outbreaks, and 0.26% in PT13a outbreaks.

## Discussion

 After a rapid increase in *S.* Enteritidis infection rates during the 1980s, the rate of sporadic cases and number of outbreaks are declining overall. Evidence of this decline has been demonstrated through passive surveillance for sporadic cases identified through the national *Salmonella* surveillance system and the number and size of *S.* Enteritidis outbreaks that occurred throughout the United States. Success has been regional, with the greatest reductions occurring in the Northeast. One of the most notable features of this decline has been a reduction in the number of outbreaks of *S.* Enteritidis infection and outbreak-associated deaths occurring in hospitals and nursing homes during the 1990s. Although the exact reason for the overall decline in infections and outbreaks has not been proven, the many prevention and control measures implemented throughout the 1990s likely played a role. These measures include tracebacks, on-farm testing, quality assurance programs, regulations regarding refrigeration, educational messages for safe handling and cooking of eggs, and enhanced surveillance.

 Tracebacks have been important in identifying farms associated with outbreaks and in tracing the spread of *S.* Enteritidis. A study of tracebacks completed in response to egg-associated outbreaks in the early 1990s showed that *S.* Enteritidis was isolated from the environment of all 14 implicated flocks tested (*9*). Diversion of eggs from *S.* Enteritidis–positive farms to pasteurization or hard-cooking facilities has also shown to be an effective strategy in reducing the number of human cases (*10*).

 Quality assurance programs, first implemented in the Northeast in the early 1990s, have been essential in monitoring and controlling the spread of *S.* Enteritidis. A USDA survey found that 56% of egg-layer farms in 15 states participated in a quality assurance program and that the number of farms routinely testing for *S.* Enteritidis had increased from 16% in 1994 to 58% in 1999 (*15*). Other important on-farm interventions include flock-based control methods such as purchasing replacement chicks from *S.* Enteritidis–negative breeders, switching to a more *S.* Enteritidis–resistant breed of layer flock, and/or the use of vaccines in commercial pullet flocks.

 Ensuring that eggs are fresh and that they are transported and stored properly are crucial steps in reducing illness. A risk assessment estimated that refrigerating eggs immediately after packing or laying could reduce *S.* Enteritidis illness by 8% and 12%, respectively (*16*). In August 1999, the USDA issued regulations stating that eggs packed for the consumer be stored and transported at a temperature of no higher than 45°F (7.2°C) and that containers for consumers be labeled to indicate that refrigeration is required (*17*). A 2000 Food and Drug Administration (FDA) rule also requires refrigeration of eggs offered for sale at retail stores (*18*). In addition, 17 states required an expiration or “sell-by” date on egg cartons in 1999 (*19*). Additional measures, such as in-shell egg heat pasteurization and irradiation, are currently available, although the effects of these on preventing human infections have not yet been measured.

 Consumers and food service workers can prevent many human infections by handling and cooking eggs properly. A recent FDA rule requires that a safe handling statement be put on all cartons of shell eggs that have not been treated to destroy *Salmonella* (*20*). This statement explains that illness from bacteria can be prevented by keeping eggs refrigerated, by cooking eggs until the yolks are firm, and by thoroughly cooking egg-containing foods. The FDA Model Food Code 1999 advises against pooling of eggs and recommends that pasteurized eggs or egg products be substituted for raw shell eggs in the preparation of foods that are not cooked (*21*). The decrease in healthcare-associated deaths may be a response to the reduction in pooling of eggs, more widespread use of pasteurized eggs, and the increased education of food workers in these facilities. This decline shows that cases of S. Enteriditis infection were prevented in persons at highest risk for serious complications from *S.* Enteritidis infection, in particular, the elderly and persons with weakened immune systems.

 Continued surveillance and outbreak investigations may help identify new vehicles of *S.* Enteritidis infection. Although a known risk factor in Europe, poultry has not previously played a large role in such infections in the United States. Our surveillance showed that in outbreaks with a known vehicle that did not contain eggs, poultry was the most common food vehicle. A study of sporadic *S.* Enteritidis infections in the United States implicated chicken as a risk factor for *S.* Enteritidis illness (*22*). In addition, a USDA survey of large production facilities that use Hazard Analysis and Critical Control Points (HACCP) plans found *S.* Enteritidis on 2.4% of broilers at slaughter (*23*). In 2000, sprouts and unpasteurized juice were identified as food vehicles in two *S.* Enteritidis outbreaks (CDC, unpub. data). These findings suggest that “new” vehicles may begin to play a larger part in future outbreaks.

 Phage typing has proved to be a useful method for monitoring the spread of various strains in the United States over time. The appearance of *S.* Enteritidis PT4 in Europe led to a dramatic increase in the number of human *S.* Enteritidis infections. In the United States, PT8 and PT13a were the predominant phage types during the 1980s, and *S.* Enteritidis PT4 was not associated with domestically acquired *S.* Enteritidis infections. In 1993, the first U.S. outbreak of *S.* Enteritidis PT4 infection occurred in Texas (*24*), and over the next 3 years, PT4 caused human illness in California, Utah, Nevada, Arizona, and Hawaii. The introduction of PT4 in Utah caused a five-fold increase in human *S.* Enteritidis cases within 6 months (*25*). This paralleled the introduction of *S.* Enteritidis PT 4 into southern California in 1994, where it also caused a substantial increase in human illness (*7*). Since then, the number of outbreaks in the Pacific region has increased greatly. *S.* Enteritidis PT 4 has also been isolated from eggs and the farm environment of laying flocks implicated as sources for human outbreaks (*25*). Despite the increase in cases and outbreaks caused by PT4, severity of disease does not appear to be increasing. Hospitalization and death rates seen in PT4 outbreaks do not differ from those of other phage types. Understanding the spread of *S.* Enteritidis PT4 and other emerging phage types may give new clues to the prevention of human illness.

 Despite these declines in *S.* Enteritidis infection, much more remains to be done. Cases of infection identified through outbreak investigations represent only a small fraction of reported infections. It is estimated from FoodNet4 data that for every case of *S.* Enteritidis infection identified as many as 37 go unrecognized (D. Voetsch et al, unpub. data). So, in 1999, as many as 200,000 cases may have occurred, of which only 5,343 were reported to CDC (*1*). To return to the 1976 baseline rate of 0.55 *S.* Enteritidis infections per 100,000 persons, a further 72% reduction in reported infections is required. A risk assessment conducted by USDA suggests that a broad-based policy is likely to be more effective in eliminating egg-associated *S.* Enteritidis illness than a policy directed solely at one stage of the continuum from egg production to consumption (*16*). To meet the challenge of further reducing such infections, the President’s Council on Food Safety announced an Egg Safety Action Plan on December 10, 1999, with the interim goal of reducing egg-associated *S.* Enteritidis illnesses by half by 2005 and eliminating them by 2010 (*26*). The plan calls for cooperation between industry, regulatory agencies, and local, state, and federal officials to implement specific controls along the entire farm to table continuum.

## Conclusion

The incidence of *S.* Enteritidis illness and the number of such outbreaks in the United States have decreased by almost 50% since the mid-1990s. The most dramatic changes are the decrease in the number of outbreaks seen in the Northeast and the reduction in numbers of outbreaks and *S.* Enteritidis–associated deaths in hospitals and nursing homes. Although the exact mechanism behind these decreases has not been explained, the reductions are likely a result of intervention programs along the farm-to-table chain. Despite these accomplishments, more work needs to be done. Further success will be measured by our ability to consistently apply and successfully monitor *S*. Enteritidis prevention and control measures along the entire farm-to-table continuum.

## References

[1] Centers for Disease Control and Prevention. Public Health Laboratory Information System. CDC *Salmonella* surveillance summaries 1976–1999. Washington: U.S. Government printing Office; 2000.

[2] Mishu B, Koehler J, Lee L, Rodrique D, Brenner F, Blake P, Outbreaks of *Salmonella* Enteritidis infections in the United States, 1985–1991. J Infect Dis. 1994;169:547–52. 8158026 10.1093/infdis/169.3.547

[3] Hickman-Brenner FW, Stubbs AD, Farmer JJ. Phage typing of *Salmonella enteritidis* in the United States. J Clin Microbiol. 1991;29:2817–23. 1757554 10.1128/jcm.29.12.2817-2823.1991 PMC270439

[4] Rampling A. *Salmonella enteritidis* five years on. Lancet. 1993;342:317–8. 10.1016/0140-6736(93)91466-Y 8101578

[5] Communicable Disease Surveillance Centre. *Salmonella* in humans. England and Wales:quaterly report. Commun Dis Rep CDR Rev. 1995;5:47.

[6] St Louis ME, Morse DL, Potter ME, DeMelfi TM, Guzewich JJ, Tauxe RV, The emergence of grade A eggs as a major source of *Salmonella* Enteritidis infections: new implications for the control of salmonellosis. JAMA. 1988;259:2103–7. 10.1001/jama.259.14.2103 3279240

[7] Passaro DJ, Reporter R, Mascola L. Epidemic *Salmonella* Enteritidis infection in Los Angeles County, California: the predominance of phage type 4. West J Med. 1996;165:126–30. 8909164 PMC1303718

[8] Henzler DJ, Ebel E, Sanders J, Kradel D, Mason J. *Salmonella* Enteritidis in eggs from commercial chicken layer flocks implicated in human outbreaks. Avian Dis. 1994;38:37–43. 10.2307/1591834 8002898

[9] Altekruse S, Koehler J, Hickman-Brenner F, Tauxe RV, Ferris K. A comparison of *Salmonella* Enteritidis phage types from egg-associated outbreaks and implicated laying flocks. Epidemiol Infect. 1993;110:17–22. 10.1017/S0950268800050639 8432319 PMC2271964

[10] Trepka M, Archer J, Altekruse S, Proctor M, Davis J. An increase in sporadic and outbreak-associated *Salmonella* Enteritidis infections in Wisconsin: the role of eggs. J Infect Dis. 1999;180:1214–9. 10.1086/314984 10479150

[11] Gast RK, Beard CW. Detection and Enumeration of *Salmonella enteritidis* in fresh and stored eggs laid by experimentally infected hens. J Food Prot. 1992;55:152–6. 31071840 10.4315/0362-028X-55.3.152

[12] Humphrey TJ, Baskerville A, Mawer S, Rowe B, Hopper S. *Salmonella enteritidis* phage type 4 from the contents of intact eggs: a study involving naturally infected hens. Epidemiol Infect. 1989;103:415–23. 10.1017/S0950268800030818 2691262 PMC2249537

[13] Martin S, Bean N. Data management issues for emerging diseases and new tools for managing surveillance and laboratory data. Emerg Infect Dis. 1995;1:124–8. 8903181 10.3201/eid0104.950403 PMC2626897

[14] Ward LR, De Sa JDH, Rowe B. A phage-typing scheme for *Salmonella enteritidis.* Epidemiol Infect. 1987;99:291–4. 3315705 10.1017/s0950268800067765 PMC2249269

[15] *Salmonella enterica* serotype Enteritidis in table egg layers in the U.S., layers ‘99. United States Department of Agriculture, Animal and Plant Health Inspection Service, Veterinary Services; 2000. [Accessed November 4, 2003] Available from: URL: http://www.aphis.usda.gov/vs/ceah/cahm/Poultry/lay99se.pdf

[16] United States Department of Agriculture Food Safety Inspection Service. *Salmonella* Enteritidis risk assessment: shell eggs and egg products. Washington: U.S. Government Printing Office; 1998.

[17] United States Department of Agriculture Food Safety Inspection Service. Refrigeration and labeling requirements for shell eggs [Final rule]. Fed Regist. 1998;63:45663–75.

[18] Food and Drug Administration. Food labeling, safe handling statements, labeling of shell eggs; refrigeration of shell eggs held for retail distribution [Final rule]. Fed Regist. 2000;65:76092–114. 11503723

[19] U.S. General Accounting Office. Food safety: U.S. lacks a consistent farm-to-table approach to egg safety. Report no. GAO/RCED-99-184. Washington: U.S. General Accounting Office; 1999.

[20] Food and Drug Administration. Food labeling, safe handling statements, labeling of shell eggs; refrigeration of shell eggs held for retail distribution [Final rule]. Fed Regist. 2000;65:76092–114. 11503723

[21] Food and Drug Administration. Food code. Washington: Food and Drug Administration; 1999. p. 47, 58, 74–5.

[22] Kimura A, Reddy S, Marcus R, Cieslak P, Mohle-Boetani J, Kassenborg H, Chicken, a newly identified risk factor for sporadic *Salmonella* Enteritidis infections in the United States: a case-control study in FoodNet sites. abstr.# 540 Fr. In: Abstracts of the 36th Infectious Disease Society of America Annual Meeting, 1998. Denver: Infectious Disease Society of America; 1998. 10.1086/381576 15095196

[23] United States Department of Agriculture, Food Safety Inspection Service. Salmonella serotypes isolated from raw meat and poultry January 26, 1998, to January 25, 1999. Available from: URL: http://www.fsis.usda.gov/OPHS/haccp/sero1yr.htm.

[24] Boyce TG, Koo D, Swerdlow DL, Gomez TM, Serrano B, Nickey LN, Recurrent outbreaks of *Salmonella* Enteritidis in a Texas restaurant: phage type 4 arrives in the United States. Epidemiol Infect. 1996;117:29–34. 10.1017/S0950268800001096 8760947 PMC2271674

[25] Sobel J, Hirshfeld AB, McTigue K, Burnett CL, Altekruse S, Brenner F, The pandemic of *Salmonella* Enteritidis phage type 4 reaches Utah: a complex investigation confirms the need for continuing rigorous control measures. Epidemiol Infect. 2000;125:1–8. 10.1017/S0950268899004057 11057952 PMC2869562

[26] President’s Council on Food Safety. Egg safety from production to consumption: an action plan to eliminate *Salmonella* Enteritidis illnesses due to eggs. Washington: President’s Council on Food Safety; 1999.

